# Tunable metasurfaces for visible and SWIR applications

**DOI:** 10.1186/s40580-019-0213-2

**Published:** 2020-01-20

**Authors:** Chang-Won Lee, Hee Jin Choi, Heejeong Jeong

**Affiliations:** 1 Institute of Advanced Optics and Photonics, Department of Applied Optics, Hanbat National University, Daejeon, 34158 Korea; 2 Department of Physics, Faculty of Science, University of Malaya, 50603 Kuala Lumpur, Malaysia

**Keywords:** Metasurface, Metamaterial, Visible, SWIR

## Abstract

Demand on optical or photonic applications in the visible or short-wavelength infrared (SWIR) spectra, such as vision, virtual or augmented displays, imaging, spectroscopy, remote sensing (LIDAR), chemical reaction sensing, microscopy, and photonic integrated circuits, has envisaged new type of subwavelength-featured materials and devices for controlling electromagnetic waves. The study on metasurfaces, of which the thickness is either comparable to or smaller than the wavelength of the considered incoming electromagnetic wave, has been grown rapidly to embrace the needs of developing sub 100-micron active photonic pixelated devices and their arrayed form. Meta-atoms in metasurfaces are now actively controlled under external stimuli to lead to a large phase shift upon the incident light, which has provided a huge potential for arrayed two-dimensional active optics. This short review summarizes actively tunable or reconfigurable metasurfaces for the visible or SWIR spectra, to account for the physical operating principles and the current issues to overcome.

## Introduction

Electromagnetic or photonic metamaterials are artificial materials made of natural metals or dielectrics so as to be specially engineered to provide new and exotic interactions between incident waves and matter. Metamaterials show phenomena that are not observed in natural or conventional materials, such as the negative refractive index [1–3], the perfect absorption [4], subwavelength focusing [5] and hyperbolically-engineered dispersion [6, 7]. Metasurface, which is the two-dimensional cousin of the metamaterial, has the thickness smaller than the wavelength of the incident light that allows control of the optical wavefront over subwavelength thicknesses [8]. Therefore, the interaction between the metasurface and the light has to be enough in order to alter the characteristics of the incident light. The characteristics of a metamaterial or a metasurface are primarily determined by its inner structure called “meta-atom” and the interaction between them. Even though meta-atom is originally defined as the unit cell in a uniformly periodic structure, many non-periodic or non-uniformly engineered meta-atoms are now available for passive metasurface applications such as lenses [9–15], axicons [16, 17], polarization converters [18, 19], and holograms [20–22].

Recent advancement in metasurfaces allows active control of light beyond manipulating the characteristics of the light under stationary platforms. Manufacturing metasurfaces becomes more viable compared to its three-dimensional cousin, because of its planar geometry and the well-established lithographic fabrication processes. The active tuning of light through a three-dimensional tunable metamaterial can be obtained by various external stimuli by electrical [23–26], mechanical [27–30], optical [31–33], thermal, or magnetic means. For mid-infrared or terahertz radiation spectra, split-ring-based, dielectric resonator-based, phase-change-materials-based, graphene-based, or liquid–crystal-based metamaterials are available, as reviewed in the previous literature [34–38]. Each control requires materials with significant optical characteristics to change accordingly.

Microscopic origin responsible for metasurface properties can be explained in terms of phase shift of incident light. The phase shift alters reflection, transmission, phase, polarization, and frequency states of the incident light. In order to gain full control of transmitted or reflected electromagnetic waves from a metasurface, it is necessary to have a full phase shift up to 2π upon incident waves. For passive metasurface with thickness *t*, typical phase shift for normal incidence after the transmission is approximated by

$$\phi = 2\pi nt/\lambda ,$$

where $\lambda$ is the wavelength of the incident wave and n is the effective refractive index of the metasurface. The phase shift of the planar-shaped metasurfaces can be limited if the used natural materials’s refractive index is not high enough. It is noteworthy that the phase distribution of modern metasurfaces is independently controlled my meta-atoms and can be either continuous and discontinuous [39]. Even though there are a number of materials showing such a controlled optical phase shifts mid-infrared or longer wavelengths, there have been quite challenging to obtain thin materials or devices for tunable metasurface in visible and SWIR regions. The difficulty comes from that natural materials, including semiconductors and conducting oxides, tend to show decreasing behavior of dielectric permittivities approaching unity, almost inversely proportional to the square of the radiation frequency (or proportional to the square of the wavelengths in a vacuum) [40]. If the dielectric permittivity of the parallel component to the incident light is close to unity, no interesting metasurface characteristics emerge.

Tunable metasurfaces for visible and SWIR spectra can be categorized, based on its controlling methods, as (1) electrically-tunable, (2) electromechanically-tunable, (3) nonlinear-optically-tunable, and (4) thermally-tunable ones, similar to the categories of three-dimensional metamaterials. There are other tuning mechanisms such as magneto-optical tuning methods and however, the workable wavelengths from other mechanisms are not currently available in the visible or SWIR light.

Electrical control on metasurface accompanies external bias potential across the entire metasurface or on part of the metamaterials nearby. The external bias reaches individual meta-atoms or a local inner structure [41]. The biasing geometries can be similar to the cases of two-terminal diodes or three-terminal field-effect transistors. Electromechanically-tunable metamaterials use electromechanical control for compressing, stretching, or pressuring to change the periodicities of meta-atoms fabricated on a flexible substrate. Likewise, nonlinear-optically-tunable metamaterials use optical pumping for dynamic control of optical properties such as optically induced transition or $\upchi^{\left( 3 \right)}$ nonlinearity. Thermally tunable metasurfaces use phase-change material, which shows dielectric permittivity changes due to its crystalline or electronic phase change according to the temperature.

So far, the most successfully engineered material in the visible or SWIR light manipulation is a liquid crystal being widely used in visual displays. However, the thickness of the liquid crystal layer has to exceed ~ 100 μm in order to gain the full 2π phase shift. The maximum modulation speed and anchoring problem are inherently limited by molecular orientation liquid crystal molecules. However, recent advancements in semiconductors, transparent conducting oxides, phase-change materials, and two-dimensional materials begin to invoke potentials to provide fast modulation tunability bandwidth exceeding 10 GHz in visible and SWIR spectra on the very thin material platform less than 10 μm [42–44]. Therefore, the development of tunable metamaterials and metasurfaces in the visible and SWIR spectra provides a great impact on optical and photonic applications with ultra-small form factors for the upcoming 4th industrial revolution. In this review, physical principles and tuning mechanisms of individual meta-atom in tunable metasurfaces are discussed with emphasis on the distinctive characteristics of the applied materials and potential applications.

## Electrically-tunable metasurfaces

Electrically-tunable metamaterials and metasurfaces for visible or SWIR spectra use local refractive index change according to charge carrier redistribution upon external perturbation. Here charge carriers can be electrons, holes, or ions. Biasing individual meta-atom results in temporally changing electromagnetic phase distribution determined by dynamically-controlled carrier concentration changes. Electric field-effect based light modulation has distinct advantages over liquid crystal-based modulation because it provides (1) fast response (typically > 1 MHz bandwidth of modulation), (2) relatively low power consumption with devices sizes smaller than submicron sizes, (3) CMOS fabrication compatibility, and (4) capability of high-density integration. Estimating carrier concentration of material is important because the redistributed carrier determines the possible phase shift range upon incident light wavelength, the ratio of polarization, and scattering direction [45].

There have been four proposed physical mechanisms based on tunable dispersion relations to design the electronically-tunable metasurfaces. (1) First, dispersion with epsilon-near-zero (ENZ) conditions for semiconducting or oxide materials has long been investigated. The ENZ condition is fulfilled when the real part of the dielectric permittivity approaches zero at a certain wavelength at the interface with adjacent materials. Therefore, the electric field virtually goes to infinity to satisfy the boundary conditions of Maxwell’s equations [46, 47]. The most intensively studied ENZ material in the visible or SWIR spectra is a transparent conducting oxide, such as indium tin oxide (ITO) and aluminum-zinc oxide (AZO). (2) The second candidate for enhancing light-matter interaction is the hyperbolic dispersion condition [6, 48]. Strong anisotropic dispersion in stacked multilayers or pillars of metallic and dielectric materials leads to field enhancement along a direction to the hyperbolic wave vector becoming imaginary. (3) Another proposed mechanism is based on altering Mie resonance condition [24, 49], however, this idea has yet been realized in the visible or SWIR spectra. (4) The most recently physical mechanism is based on dynamic control of quantum-confined confined Stark effect [50].

The ENZ dispersion condition for tunable metasurfaces can be understood qualitatively as in the following manner. The meta-atom with two or more electrodes can be regarded as a capacitor. Therefore, the carrier concentration under ENZ condition voltage can be approximately estimated by matching the driving voltage and the dielectric permittivity. The driving voltage required to reach ENZ is defined as

$${\text{V}} = N_{ENZ} d_{ACL} /{\text{C}}$$

where $N_{ENZ}$ is the free carrier concentration in the considered material at the ENZ condition and $d_{ACL}$ is the thickness of the charge accumulation layer. Because the capacitance ${\text{C}}$ is given by

$${\text{C}} = \varepsilon_{0} \varepsilon_{r} /d_{r}$$

where $\varepsilon_{0}$ is the permittivity of vacuum, $\varepsilon_{r}$ is the dielectric constant of the material, and $d_{r}$ is the thickness of the material, the electric field at ENZ condition is given by

$${\text{E}} = \frac{\text{V}}{{d_{r} }}$$

The wavelength and the angle of incidence at ENZ condition can be found by searching poles in the Fresnel formula for transmittance or reflectance. Using the Drude model, it is possible to estimate carrier concentration using this form of the electric field as an external stimulus.

**Fig. 1:**
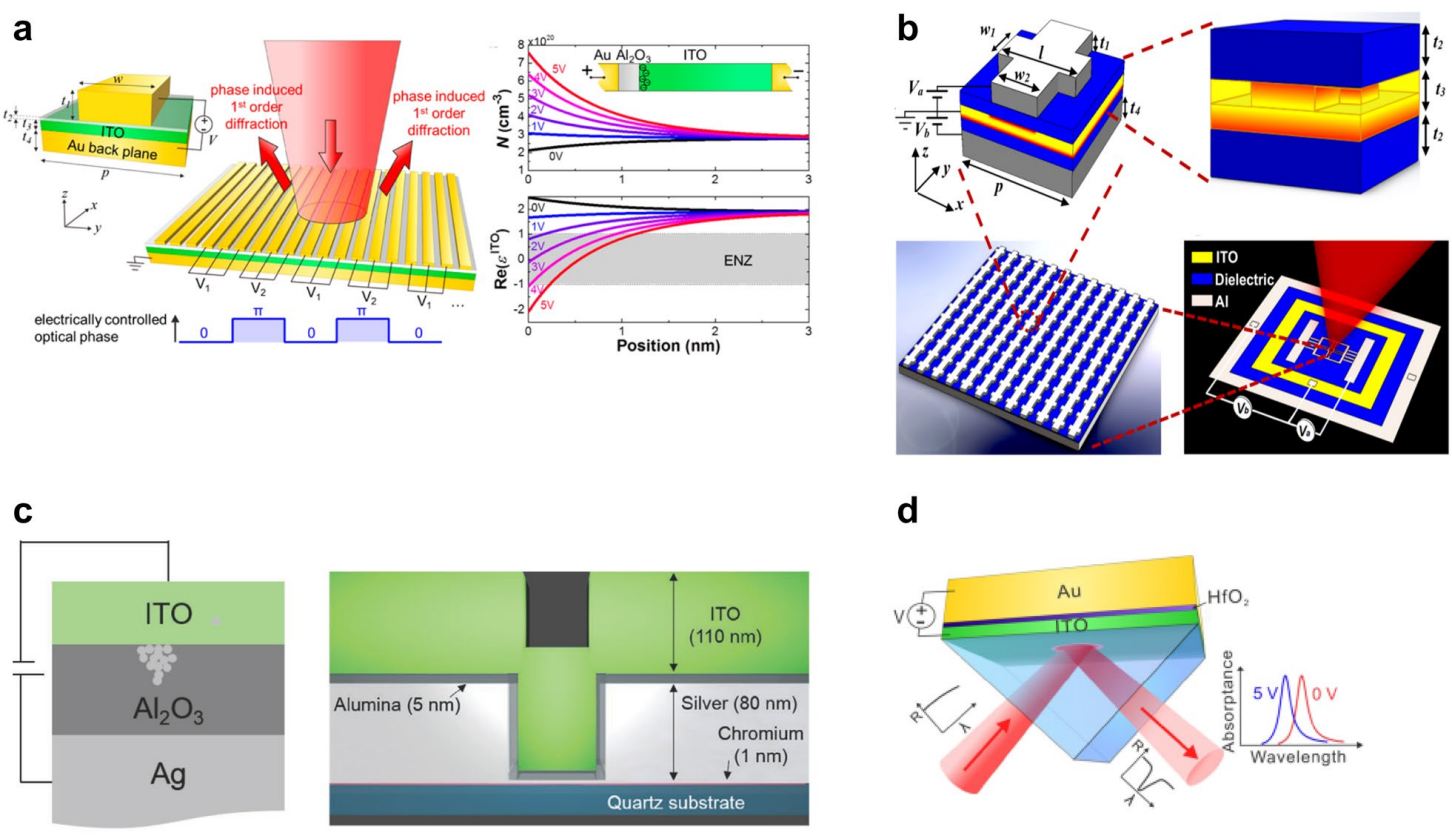
Tunable meta-atoms with transparent conducting oxides. **a** An ITO layer under external bias modulates the charge accumulation region near ENZ dispersion condition, resulting in a phase shift for the reflected light in the SWIR region [51]. Reproduced with permission from ©2016, American Chemical Society. **b** Dual gated ITO layer for enhanced phase shift up to 303^°^ at 1550 nm [52]. Reproduced with permission from ©2018, American Chemical Society. **c** Ionic-conduction-induced visible tunable meta-atom [40]. Reproduced with permission from ©2017, Wiley–VCH. **d** Oxide heterostructure for Berreman dispersion mode [53]. Reproduced with permission from ©2018, American Chemical Society

One of the noticeable metasurface structures based on electric field-effect based SWIR light modulation has been demonstrated by Huang et al. [51]. The electrically tunable metasurface consists of an electrically bus-connected one-dimensional gold nanoantenna array patterned on thin Al_2_O_3_ and ITO layers, deposited on a gold mirror as shown in Fig. 1a. Al_2_O_3_ layer, grown by atomic-layer-deposition (ALD) method, provides good thermal stability and a high breakdown field larger than 10 MV/cm [54]. Identical antenna arrays are connected either to right or left external gold connections and electrodes to permit phase and amplitude modulation by electrical gating. Oxide-based field-effect modulation consists of metal–transparent conducting oxide–metal configuration where transparent conducting oxide acts as a semiconductor. All the optical antennae have identical geometries, but applying different voltages to neighboring antennae controls the phase shift imposed by the different antennae. Each antenna was designed with a shape to impose a phase shift on the incident plane wave, which is pre-defined by the lithographic patterning process.

Each unit cell is a metal/insulating oxide/semiconductor (MOS) capacitor where the top antenna and back reflector act as capacitive electrodes. By increasing the bias voltage between the top and back electrodes, charge accumulation or depletion region which can induce the change of carrier density forms in the ITO layer, at the ITO/Al_2_O_3_ interface. This results in the modification of the complex permittivity of ITO, and in the phase shift between the reflected and incident lights. Because each antenna can be biased individually, the phase distribution across the metasurface effectively controls the overall wavefront, leading to manipulated reflected light. Also, dynamic gate voltage control enables dynamic control of the phase shifts reflected by the individual antenna, facilitating active beam directionality control or high-speed intensity modulation of the reflected light. The authors designed device structure to obtain as close ENZ condition at the interface between the ITO/Al_2_O_3_ interface as possible because ENZ leads to large electric field enhancement that occurs in the accumulation layer for short-wavelength infrared wavelengths [55, 56]. Based on the Drude model, the carrier concentration and the resultant dielectric permittivity could be estimated [45, 51, 57]. Regarding ITO as a semiconductor material with a bandgap of *E*_*bg*_ = 2.8 eV, the electron affinity of $\upchi$ = 5 eV, and effective electron mass of m* = 0.25 *m*_*e*_, the dielectric permittivity can be written as

$$\varepsilon_{ITO} \left(\upomega \right) = \varepsilon_{\infty } - \frac{{\upomega_{p}^{2} }}{{\upomega^{2} + i\upomega\varGamma }},\quad\upomega_{p}^{2} = \frac{{Ne^{2} }}{{\varepsilon_{0} m^{*} m_{e} }},$$

where *m*_*e*_ is the mass of the free electron, $\varepsilon_{\infty } = 4.2$, $\upomega_{p}$ is the plasma frequency, $\varGamma$ is the damping constant of 1.8 × 10^14^ rad Hz, *e* is the electron charge, *ε*_*0*_ is the dielectric permittivity of vacuum, and *N* is the carrier density. As a result, the estimated background carrier concentration of the fabricated ITO film ranged from *N*_*o*_ = 8 × 10^19^ cm^−3^ to 7 × 10^20^ cm^−3^ depending on the growth conditions. The accumulated carrier concentration and the real part of the dielectric permittivity as a function of the position from the Al_2_O_3_/ITO interface are shown in Fig. 1a. Under the varied gate bias from 0 V to 5 V, the electron carrier density can be increased or decreased at the Al_2_O_3_/ITO interface, resulting in the change of the real part of the permittivities from positive to negative values.

The performance of the device shows the reflectance change, defined as $\frac{\Delta R}{R} = \frac{{R\left( V \right) - R\left( {V = 0V} \right)}}{{R\left( {V = 0V} \right)}}$, almost reaches ~ 30% at 1600 nm and ~ − 20% at 1500 nm, under an applied bias of 2.5 V. The phase shift, measured by a Michelson interferometer incorporating self-phase referencing method, show increasing phase shift with applied bias. A maximum phase shift was obtained under 2.5 V gate bias around 184 ^°^. The authors also confirmed the modulation bandwidth as high as 10 MHz, mainly limited by large capacitance value owing to the large area of the antenna. The largest bandwidth with antennas of submicron could be estimated to exceed 100 GHz [51].

Shirmanesh et al. introduced a dual-gated geometry to the ITO active material to maximize phase shift, as shown in Fig. 1b [52]. This configuration allows the application of two independent gate biases between the back reflector and ITO, and between top antenna and ITO, enabling larger phase tunability compared to the single gate. For enhanced field between the gate dielectric/ITO interfaces, they also incorporated ENZ condition at the interface between the gate dielectric and ITO layers to design the structure, leading to a 5 nm-thick ITO layer. For the top gate, a fishbone structure was introduced. Another improved feature is ALD-grown 9.5 nm-thick Al_2_O_3_/HfO_2_ nanolaminates (HAOL) as gate dielectrics. This hybrid material improved gate-dielectric material enabled larger DC permittivity up to 22, which is close to the value of HfO_2_ (~ 25). Two independent gating configurations were tested: (I) The gates are applied with V_0_ while the ITO layer is kept ground. (II) The top gate is applied with +V_0_ and the bottom gate is applied with −V_0_, while the ITO layer is kept ground. In the case (I), the authors could achieve significant reflectance change and phase shifts, from − 212 ^°^ to + 91 ^°^, up to 303 ^°^ phase shift at 1550 °nm. This maximum phase shift was obtained when the injected carrier in the entire ITO layer is unipolar.

Optical modulation in visible spectra was demonstrated by Thyagarajan et al., using ionic conduction change [40]. In this work, an Ag/Al_2_O_3_/ITO heterostructure, which allows Ag diffusion into Al_2_O_3_ and ITO layer, significantly alters dielectric characteristics, resulting in large phase shifts in the visible. Figure 1c shows the device structure with Ag ion diffusion. Like resistive switching devices, these ions diffuse repetitively in and out in the oxide layers depending on the applied bias. When a positive voltage is applied to the Ag electrode, the Ag ions migrate to the opposite inert electrode (ITO) and form Ag filaments in the Al_2_O_3_ layer. The growth of Ag nanoparticles in the ITO with increasing applied bias voltages alters optical reflectance and transmittance. Owing to the large device area up to 1 mm × 1 mm, RC time delay limits the modulation frequency up to 600 Hz with a 20% on–off ratio of normalized reflectance. One of the concerns of the ionic diffusion is the endurance of the device, which often leads to reduced functionality in time, as common as in the resistive random-access memory (ReRAM).

Anopchenko et al. demonstrated electrical tuning of reflectance and perfect absorption from multilayer stacks of ITO layers with a gradient of electron densities near ENZ condition as shown in Fig. 1d [53]. The gate bias up to 5 V was applied, similar to a conventional metal–oxide–semiconductor transistor, to make incident light absorbed by matching Berreman and ENZ modes to perfection absorption condition with an optimized thickness of ITO layer. For better absorption, the authors introduced a 5 nm-thick HfO_2_ layer, which leads to near unity absorption with less than 16 nm of oxide multilayers.

**Fig. 2:**
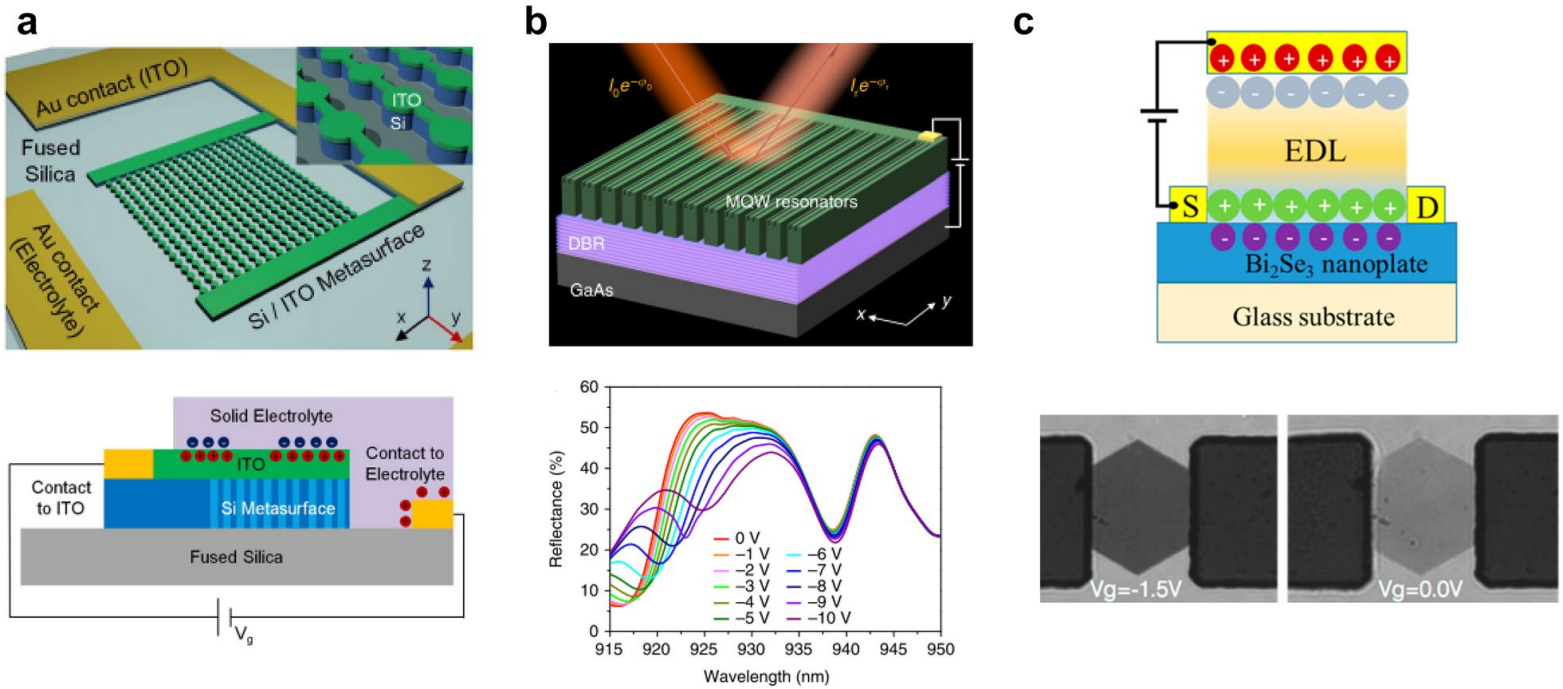
Semiconducting material based tunable metasurfaces. **a** ITO-Si nanoantenna heterojunction for tunable metasurface. Si nanoantennas support optical resonance and electrical bias simultaneously [58]. Reproduced with permission from ©2018, Optical Society of America. **b** GaAs-based semiconductor heterostructure for tunable quantum-confined Stark effect [50]. Reproduced with permission from ©2019, Springer Nature. **c** Bi_2_Se_3_-based tunable absorber [59]. Reproduced with permission from ©2015, American Chemical Society

Howes et al. combined ENZ condition of thin dielectric resonators to realize Huygens-mode tunable metasurface, as shown in Fig. 2a [58]. The field enhancement in an ITO layer sandwiched by Si resonator and solid electrolyte can be largely modulated owing to charge accumulation near ENZ condition. The on-state transmittance was 70% in the SWIR and the modulation depth reaches 31%.

Wu et al. introduced another type of electro-optic effect, based on the quantum-confined Stark effect in a III–V multiple quantum well, as shown in Fig. 2b [50]. The modulated dispersion coupled both with Mie resonance and with guided-mode allows 270% relative reflectance modulation depth and a phase shift up 70 °. At 914 nm, the tunable metasurface works as an electrically-driven beam steering device for remote sensing applications.

Two-dimensional materials, namely 2D materials, which comprises of atomically thin layers, have recently significant attention owing to optically tunable phase transitions [60], and intervalley transitions [61, 62]. Since the discovery of graphene’s gate-tunable optical properties has been known, various types of 2D materials, especially transition metal dichalcogenides (TMDs) have been intensively studied. TMDs show strong excitonic properties and distinctive valley-dependent dispersion, which is originated pseudospin splitting induced by the magnetic field from Berry curvature in momentum space. In the lack of inversion symmetry, the carriers in the valleys can be selectively excited or detected by circularly-polarized light (chiral behavior) [63, 64], Because the carriers in 2D materials are strongly affected by electric gating field as well, the dielectric permittivities, especially chirality dependent Kerr permittivities also undergo strong change due to electrostatic gating. A recent study shows TMD materials can also serve optically active materials for photodetectors and photon emitters [63].

Even though 2D materials show tunability for GHz or THz frequencies by electro-optic perturbation or by nonlinear responses [65], there are few demonstrations for the spectral region of visible and SWIR light. 2D materials do not have enough free carrier concentrations to directly gate through dielectric space. Therefore, ionic liquid gating was introduced to increase carrier concentration and Fermi level. Liu et al. demonstrated ionic liquid gating across Bi_2_Se_3_ to show a great change in transmittance in the visible, as shown in Fig. 2c [59]. The transmittance varies over 60% in the visible with increasing gate voltage from − 1.5 V to 1.5 V. They figured out the dynamically tunable optical properties of Bi_2_Se_3_ is originated from optical bandgap due to the change in free-electron concentration (Burstein-Moss shift). They also performed similar experiments using MoSe_2_. However, MoSe_2_ rather show ambipolar tunability, which implies both electron and hole concentration affects optical property due to the applied bias polarity.

One of the problems in the classical ENZ model is the fixed or overestimated value of dielectric permittivity under external stimuli. If carrier concentration is redistributed, the real part of the dielectric permittivity $\varepsilon_{TCO}$ should either be increased or decreased depending on the sign of the bias voltage. To account for this problem, a quantum mechanical model has been recently suggested. The quantum mechanical model is based on the density gradient current, with the expression for electron current given by

$$\overrightarrow {{J_{n} }} = - \mu k_{B} {\text{T}}\nabla n - \mu n\nabla \left( {\varPhi + \varLambda } \right)$$

Here, $n$ is the electron or hole concentration, ${\text{T}}$ is the lattice temperature, $\varPhi$ is the potential including both band edges and electrostatic potential. The last term is the quantum-mechanically-corrected potential given by

$$\varLambda = - \frac{{\gamma \hbar^{2} }}{6m}\frac{{\nabla^{2} \sqrt n }}{\sqrt n },$$

where γ is a fit factor, m is the carrier effective mass, and n represents electron or hole concentration as appropriate [66, 67]. The quantum model can be solved by numerical calculation or commercial software package to extract accumulated charge concentration.

The classical model and the quantum model use different boundary conditions, which leads to a significant difference in tuning range and charge concentration. Depending on the geometry, the quantum model and the classical model may produce two orders of magnitude absorption rate difference in dB/μm scale [67]. The quantum mechanical model tends to produce smaller values of accumulated charge concentration under the same external bias condition. The discrepancy in the classical Drude model and the experimental results lies in the fact that the dielectric constants of the material in the structure are actually altered due to nonequilibrium charge redistribution. Nevertheless, it is undeniable that the classical model is more convenient for the initial design of the tunable meta-atom structure.

One of the main advantages of electrically-tunable metasurfaces is that the shape of meta-atoms do not change.

## Electromechanically-tunable metasurfaces

Tunable metamaterials can be made by dynamic structural changes by the external stimuli altering the size, the shape of, and distance between meta-atoms. These mechanical stimuli can be attained by a controllable actuation of the sub-wavelength structure. However, the realization of these structures for visible or SWIR spectra is expensive due to complex submicron fabrication and mechanical endurance has to be ensured to gain popularity like a pixelated micro-electro-mechanical system (MEMS) mirror arrays.

In a practical way, manufacturing arrays of resonators on stretchable elastomeric substrates offer a dynamic tuning of the optical metamaterials. The flexible elastomeric substrate can achieve large tuning ranges and relative ease of fabrication. Because stretching or shrinking elastomeric substrates has restoration and cycling problem, the geometric design has been carefully chosen to keep linear response and restoration under repetitive stimuli.

**Fig. 3:**
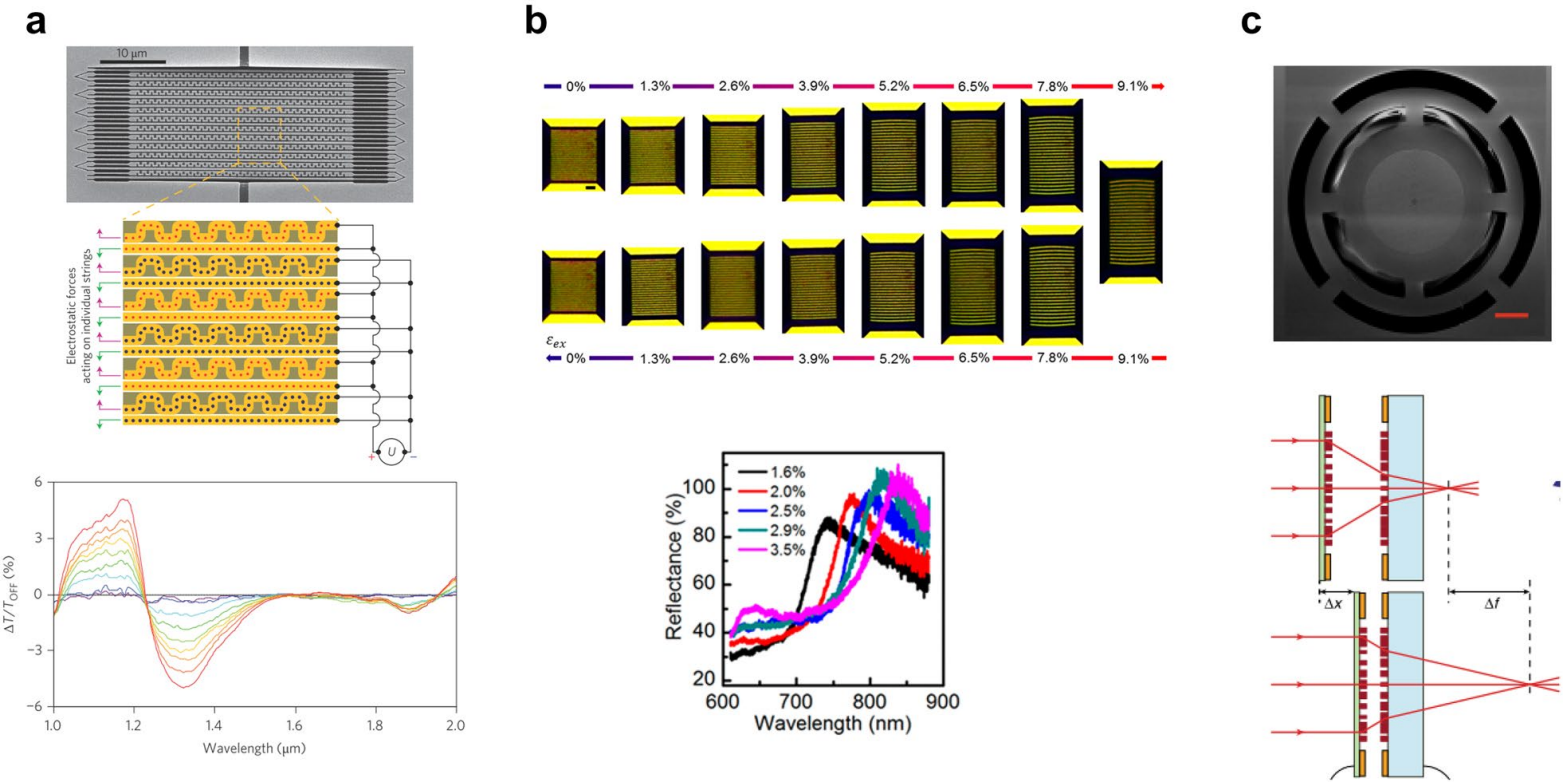
Electromechanically tunable metasurfaces. **a** Tunable microbeam array actuated by electrostatic force [28]. Reproduced with permission from ©2013, Springer Nature. **b** Strain-induced tunable grating on a PDMS substrate [68]. Reproduced with permission from ©2018, American Chemical Society. **c** Micro-electro-mechanical-system-based metalens doublet [30]. Reproduced with permission from ©2018, Springer Nature

Ou et al. demonstrated electromechanically-driven metasurface working in SWIR spectra, as shown in Fig. 3a [28]. The metasurface is fabricated by focused ion beam milling on a 50-nm-thick silicon nitride membrane. When a bias ~ 3 V is applied, the strings in the metasurface are exposed to electrostatic force, which leads to string fields in the gaps between them. As a result, the transmittance could be modulated by 5% in SWIR. The modulation depth was changing as a function of the modulation frequency.

Chen et al. demonstrated a tunable plasmonic lattice grating patterned on a flexible and stretchable PDMS substrate as shown in Fig. 3b [68]. The tunable plasmonic lattice shows the linear and reversible optical response of metasurface with little hysteresis. It shows the geometrical structure with a pair of tapered microrod at the end of plasmonic lattice grating, realizing tailorable strain amplification. The fabricated metasurface shows linear mechanical response under external strain varying 0% – 10%, while reflectance spectra modulation depth reaches 40% by external strain change from 1.6% to 3.5%. The surface plasmon resonance shifts approximately 80 nm in the visible at ~ 780 nm under the same strain variation.

Arbabi et al. demonstrated a focal-length tunable lens using a pair of metalens as shown in Fig. 3c [30]. The metalens is based on the high-contrast dielectric transmit arrays. One metalens is fabricated on a fixed glass substrate whereas the other metalens is fabricated on a movable SiN membrane. The doublet shifts the focal length up to 30 μm around 800 nm wavelength, only by moving one metalens of 1 μm. Higher bias voltage shifts the focal length up to 80 μm.

## Reconfigurable metasurfaces based on optical nonlinearity

The properties of light in metasurfaces can be controlled dynamically by means of optical pumping. An optical tuning can be achieved by inserting an active layer that responds to the pumping light in a metallic or dielectric nanocavity.

**Fig. 4:**
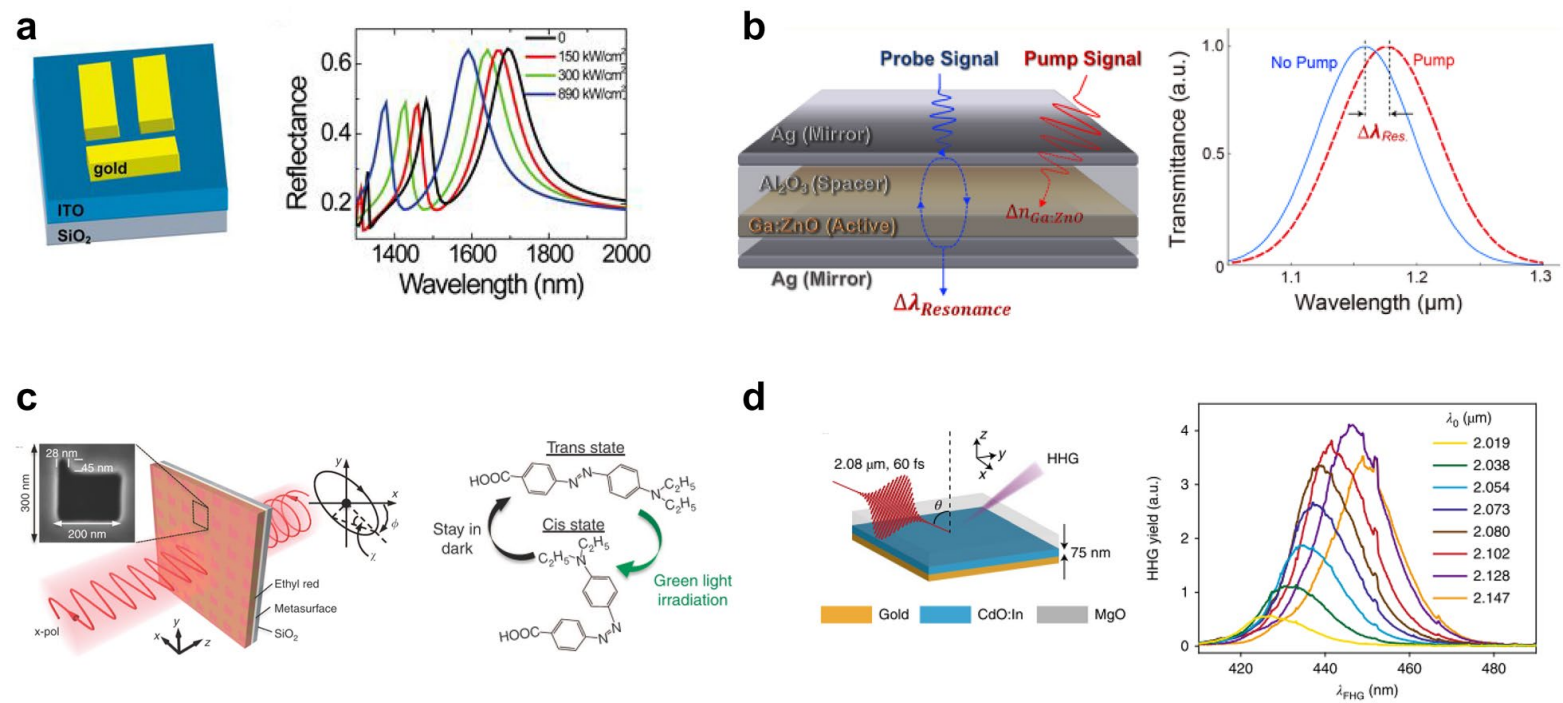
Nonlinear-optically tunable metasurfaces. **a** Plasmonic-antenna assisted tunable meta-atom by Kerr nonlinearity [69]. Reproduced with permission from ©2013, Springer Nature. **b** Optical pumping leads to Fabry-Pérot resonance shift owing to the large nonlinearity of a nanocavity [70]. Reproduced with permission from ©2018, American Chemical Society. **c** Polarization converter based on molecular transformation [71]. Reproduced with permission from ©2017, Springer Nature. **d** Fifth harmonic generation from a hybrid oxide heterostructure near ENZ condition [72]. Reproduced with permission from ©2019, Springer Nature

Zhu et al. realized plasmon-induced nonlinear tunable transparency using gold meta-atom on top of a thin polycrystalline ITO layer, as shown in Fig.4a [69]. The Kerr nonlinear index of refraction is given by

$${\text{n}} = n_{0} + n_{2} I = n_{0} + \frac{{3{\text{Re}}\left( {\chi^{\left( 3 \right)} } \right)}}{{4{\text{c}}\varepsilon_{0} n_{0}^{2} }}I_{0},$$

where $n_{0}$ and $n_{2}$ are linear anthe d nonlinear refractive index of ITO, ${\text{Re}}\left( {\chi^{\left( 3 \right)} } \right)$ is the real part of the third-order nonlinear susceptibility of the ITO, $\varepsilon_{0}$ is the permittivity of the vacuum, and ${\text{c}}$ is the light velocity in the vacuum. The plasmonic resonances of the meta-atom lead to superradiance and subradiance depending on the pump laser intensity. This effect leads to an optical transparency window shift to the short-wavelength direction in SWIR.

Kim et al. demonstrated optically tunable metasurface made of a metal–insulator-metal (Ag-Al_2_O_3_-Ag) nanocavity with a 70 nm-thick Ga:ZnO layer as an active layer, as shown in Fig. 4b [70]. Harnessed by fast switching mechanism of optical pumping, sub-picosecond switching modulation speed and 80% of modulation depth was obtained by sub 10 mJ/cm^2^ pumping fluence level. Under degenerate optical pumping, a 15 nm transmittance redshift of Fabry–Pérot resonance shift was obtained in the SWIR spectrum excited near the ENZ wavelength.

Ren et al. demonstrated a polarization-state-switching metasurface made of a plasmonic structure with isomeric ethyl-red polymers, as shown in Fig. 4c [71]. The structure consists of a 100-nm-thick gold film on a 500-μm-thick fused quartz substrate (SiO_2_) and an ~ 300 nm ethyl-red polymer layer thereon. The gold film forms a periodic array of L-shaped slots to construct metasurfaces. The polarization tuning is achieved by irradiating the green light (532 nm) that leads to change in the isomeric state of ethyl-red, that is, from trans-state to cis state which can efficiently modify the refractive index of the polymer layer. Hence the coupling between the resonant plasmonic modes and isomeric state of ethyl-red leads to polarization control of metasurfaces. When the control light is present, the refractive index decreases as ethyl-red changes to cis state, resulting in a blue shift of polarization azimuth rotation angle ϕ and ellipticity angle χ. As a result, 80% of modulation depth at 6 Hz. The switching speed is inherently limited by the recovery time from the isomerization process.

Yang et al. demonstrated visible high-harmonic generation from In-doped CdO as shown in Fig. 4d, using epsilon-near-zero condition [72]. Even though the ENZ condition is satisfied at 2250 nm, the strong field enhancement at the boundary between In-doped CdO and MgO boosts the harmonic generation, resulting in visible light generation in the fifth order. The authors attributed the origin of harmonics generation to the photo-induced electronic temperature elevation in the conduction band by comparing a relaxation model with the experimental observation of decay time.

## Thermally tunable metasurfaces based on phase change materials

Phase-change materials show near-unity refractive index change as the structural or electronic phase changes across a critical temperature. Since the application of digital versatile disc (DVD), germanium-antimony-tellurium (GST) with a crystallization temperature T_c_ of ~ 433 K (160 °C) and a melting temperature of ~ 873 K (600 °C) has long been used as a common phase change material. For the SWIR spectra application, vanadium dioxide (VO_2_) shows a hysteretic structural phase transition from the monoclinic phase to the tetragonal phase when the temperature rises above from 340 K (67 °C). Because the temperature could be risen by electrical current pulse injection, these two materials have been intensively investigated for tunable metasurface applications.

**Fig. 5:**
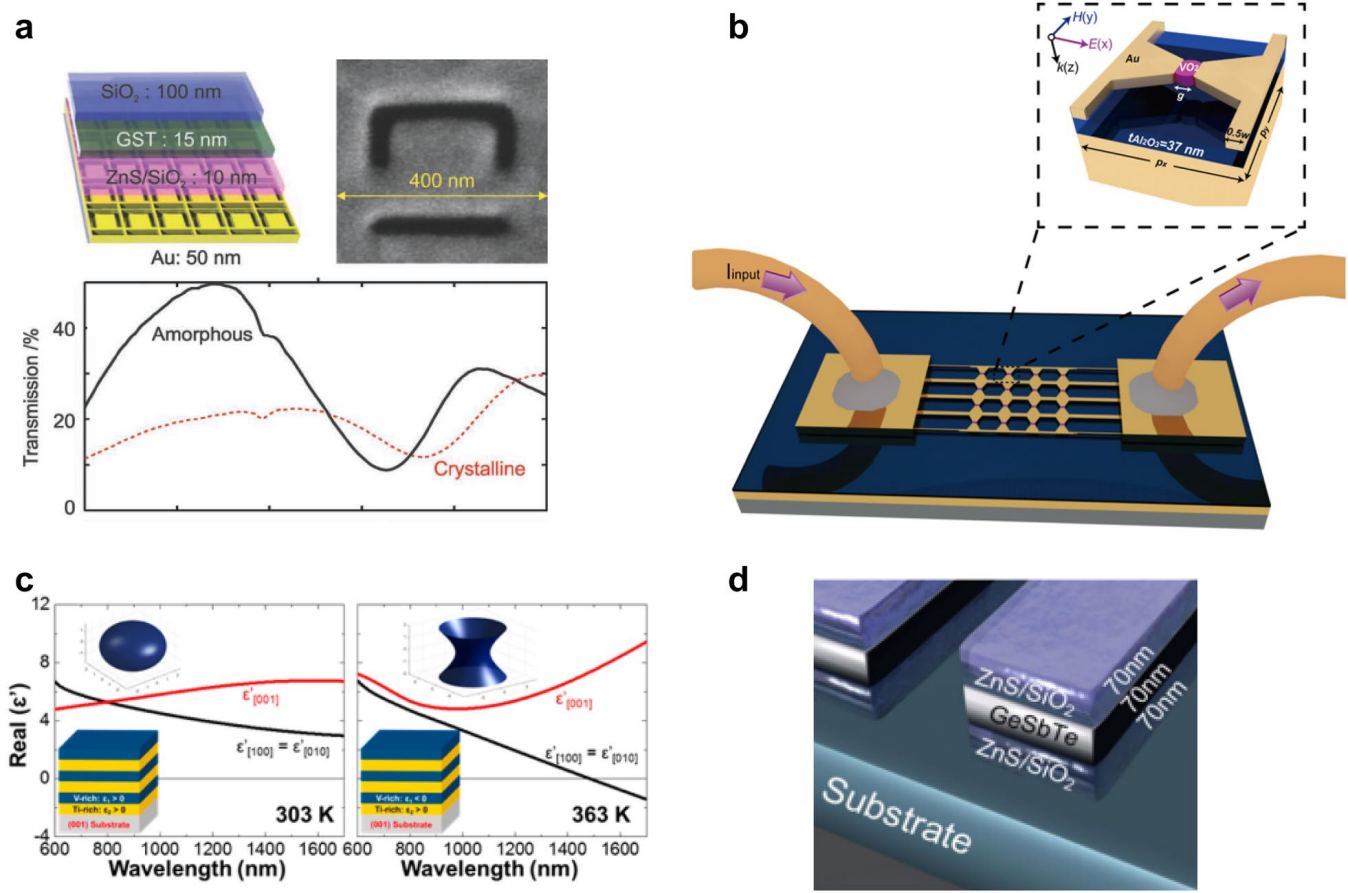
Phase-change-material-based tunable metasurfaces. **a** Optically tunable thermal metamaterial based on GST [73]. Reproduced with permission from ©2013, WILEY–VCH. **b** Meta-atom made of a bow-tie-shaped unit cell that has a small amount of the VO2 [74]. Reproduced with permission from ©2017, American Chemical Society. **c** Metal-dielectric heterostructure for hyperbolic dispersion under thermal modulation [75]. Reproduced with permission from ©2016, American Chemical Society. **d** UV light-tunable meta-atom made of GST sandwiched by ZnS/SiO_2_ layers [76]. Reproduced with permission from ©2019, American Chemical Society

Gholipour et al. demonstrated all-optical bidirectional metasurface based on the GST as shown in Fig. 5a [73]. A 15 nm-thick GST layer is sandwiched between SiO_2_ and ZnS/SiO_2_ layers. Plasmonic thin trenches made of 50-nm-thick Au layer support plasmonic resonance and enhanced photoabsorption for a temperature change of GST. As the phase of the GST layer changes from the crystalline phase to the amorphous phase, the transmission at SWIR spectra is enhanced from 20% to 40%. The temperature control of the GST layer is provided by a temporally-modulated light to morph phases between amorphous one and crystalline one.

Zhu et al. demonstrated electrically-controllable metasurface based on the phase change of VO_2_ as shown in Fig. 5b [74]. VO_2_ is laterally sandwiched by plasmonically structured Au electrodes and the modulated voltage pulse train changes the phase of VO_2_ between amorphous and crystalline phases. As a result, modulation depth up to 33% at the SWIR spectrum could be obtained with sub 10 ms response time.

Chen et al. demonstrated temperature-dependent tunable metamaterial based on vanadium dioxide(VO_2_)-titanium dioxide(TiO_2_) multilayer as shown in Fig. 5c [75]. The VO_2_–TiO_2_ multilayer shows dispersion relation change from the elliptic form to hyperbolic form as the temperature increases over the critical temperature of VO_2_ around 325 K. As a result, the real part of the dielectric permittivity of the composite metamaterial measured by spectroscopic ellipsometry changes at the SWIR spectrum.

Gholipour et al. demonstrated an optically-tunable metasurface, based on a patterned GST heterostrutcture with ZnS/SiO2 (Fig. 5d), showing tuning capability for ultraviolet (UV) and visible spectra [76]. GST shows a significantly low refractive index at the UV spectrum, as low as 1.07 at 245 nm in the crystalline phase. Also, an amorphous composite of zinc sulfide and silica with a 1:9 atomic  % ratio shows almost constant refractive index 2.4 in the UV and visible. By optical pumping, the phase changes of the GST layer leads to reflectance modulation depth over 10%.

## Conclusion and outlook

We have briefly summarized recent advances in tunable metasurfaces in the visible and SWIR, focusing particularly on available tuning mechanisms. Despite all the advancements made in the past few years, we have witnessed there are quite a number of challenges to overcome. One of the challenges in the material point of view is that the natural materials in the visible do not have enough refractive indices. This limits the minimum thickness of the materials to allow enough phase shifts originated from the electric field, electrical power to change phase or thermal or optical power. From fundamental point-of-view, tunable metasurfaces also have similar challenges as passive metasurfaces such as polarization-control, chromatic aberration, large-deflection angle, high efficiency, and the number of available degree of freedoms to solve these issues in single surface or a specific volume [8].

These problems are also related to the problems in unresolved practical issues to allow high-volume, low-cost metasurface devices. Each mechanism has clear advantages and disadvantages to meet the needs of highly pixelated tunable optical and photonic devices in visible and SWIR spectra. For thorough control of the tunable meta-atoms, individual excess to meta-atom and the required integrated biasing circuit, and simultaneous and locked operation of multiple meta-atoms are required. Also, the endurance of meta-atoms under repetitive biasing or optical stimuli and sufficient modulation depth and bandwidths to meet the full 2π phase shift in the target spectra should be overcome.

Despite the difficulties in realizing practical, real-life tunable metasurface devices, we witness continuous efforts to overcome the challenges. We recently noticed different class of tuning mechanism, based on of time-domain-control of metasurfaces, different from nonlinear-optical tunable metasurfaces [77, 78]. Nonconventional metasurfaces, based on non-Hermitian coupling, topological, non-local, and quantum–mechanical interactions are also actively studied [79]. Not to mention the importance of the new class of tuning mechanisms, we expect new tuning mechanisms could revolutionize optics and photonics beyond conventional diffractive optics and electromagnetics in the tunable metamaterials for the visible or SWIR spectra. Even though developing highly integrated tunable optoelectronic or photonic devices and systems with small form factor is a formidable task, we believe the advancement of technology will be able to bring us a new powerful tool for the development of full-random-accessible meta-atoms in a metasurface platform and to find new entrepreneur applications with tunable optical metamaterials and metasurfaces.

## Data Availability

Data sharing is not applicable to this article as no datasets were generated or analyzed during the current study.

## Abbreviations

SWIR: short-wavelength infrared
LIDAR: light detection and ranging
ENZ: epsilon-near-zero
ITO: indium tin oxide
AZO: aluminum-zinc oxide
ALD: atomic-layer-deposition
MOS: metal/insulating oxide/semiconductor
HAOL: Al_2_O_3_/HfO_2_ nanolaminates
TMD: transition metal dichalcogenide
MEMS: micro-electro-mechanical system
DVD: digital versatile disc
GST: germanium-antimony-tellurium
UV: ultraviolet
